# 

**Published:** 1911-01-07

**Authors:** 


### CONTENTS.

| | PAGE |
|---|---|
| **Medical Education in the United States** ... ... | 427 |
| **Clothing for the British Climate** ... ... ... ... ... | 428 |
| **Annotations—** | |
| "Grinding" in Ireland – Motherliness—The New Year Honours | 429 |
| **Medical Opinion and Movement** ... ... ... ... ... | 430 |
| **Hospital Clinics—** | |
| Drug Eruptions: their Nature and Varieties. By J. H. Stowers, M.D. ... ... ... ... ... ... ... ... | 433 |
| **Special Articles—** | |
| [illegible] Dioxydiamidoarsenobenzol ... ... ... ... ... ... | 437 |
| Money, Mind, and Molars ... ... ... ... ... ... | 438 |
| **Surgery—** | |
| The Operative Treatment of Fractures ... ... ... ... ... | 439 |
| **News and Events of the Week** ... ... ... ... ... | 441 |
| **Appointments and Resignations** ... ... ... ... ... | 442 |
| **Parliament and Publications** ... ... ... ... ... | 442 |
| **Obituary** ... ... ... ... ... ... ... ... ... .. | 442 |
| **The Week's Work—** | |
| Hospital Administration at the Cape—The Medical Point of View—The Cape Resolutions—The Question of Indigency—Hospitals and School-Children—School-Children and Out-patient Departments — The Future — This Week's Drug Market ... ... ... ... ... ... ... ... ... ... | 443 |
| **Hospital Architecture and Construction** ... ... | 445 |
| **Jottings** ... ... ... ... ... ... ... ... ... ... | 446 |
| **Gifts and Bequests** ... ... ... ... ... ... ... | 446 |
| **Special Institutional Article—** | |
| Hospital Administration and Organisation in South Africa ... | 447 |
| **Institutional Notes and News** ... ... ... ... | 450 |
| **The Hospital World** ... ... ... ... ... ... ... | 452 |
| **Editor's Letter-Box—** | |
| Hospital Relief in Holland ... ... ... ... ... ... | 452 |
| **New Appliances and Things Medical** ... ... ... | 453 |
| **The Week's Novelties** ... ... ... ... ... ... | 454 |

